# Resilience and Posttraumatic Growth after Burn: A Review of Barriers, Enablers, and Interventions to Improve Psychological Recovery

**DOI:** 10.3390/ebj3010009

**Published:** 2022-02-09

**Authors:** Alix Woolard, Indijah Bullman, Amira Allahham, Treya Long, Helen Milroy, Fiona Wood, Lisa Martin

**Affiliations:** 1 Telethon Kids Institute, Perth Children’s Hospital, 15 Hospital Ave, Nedlands, WA 6009, Australia; alix.woolard@telethonkids.org.au (A.W.); indijah.bullman@telethonkids.org.au (I.B.); helen.milroy@uwa.edu.au (H.M.); 2 Burn Injury Research Unit, University of WA, 35 Stirling Highway, Crawley, WA 6009, Australia; amira.allahham@research.uwa.edu.au (A.A.); treya.long@health.wa.gov.au (T.L.); fiona.wood@health.wa.gov.au (F.W.); 3 Fiona Wood Foundation, 11 Robin Warren Dr, Murdoch, WA 6150, Australia; 4 Burn Service of Western Australia, Burns Unit, Level 4, Fiona Stanley Hospital, 102–118 Murdoch Drive, Murdoch, WA 6150, Australia

**Keywords:** burn, posttraumatic growth, PTG, review, resilience

## Abstract

Burn injuries are traumatic experiences that can detrimentally impact an individual’s psychological and emotional wellbeing. Despite this, some survivors adapt to psychosocial challenges better than others despite similar characteristics relating to the burn. Positive adaptation is known as resilience or posttraumatic growth, depending on the trajectory and process. This review aimed to describe the constructs of resiliency and growth within the burn injury context, examine the risk factors that inhibit resilience or growth after burn (barriers), the factors that promote resilience or growth after burn (enablers), and finally to assess the impact of interventions that have been tested that may facilitate resilience or growth after burn. This review was performed according to the recently updated Preferred Reporting Items for Systematic Reviews and Meta-Analyses (PRISMA) Guidelines. An electronic search was conducted in November 2021 on the databases PubMed, Medline (1966-present), Embase (1974-present), PsycINFO for English-language peer-reviewed academic articles. There were 33 studies included in the review. Findings were mixed for most studies; however, there were factors related to demographic information (age, gender), burn-specific characteristics (TBSA, time since burn), person-specific factors (personality, coping style), psychopathology (depression, PTSD), and psychosocial factors (social support, spirituality/religion, life purpose) that were evidenced to be related to resilience and growth. One qualitative study evaluated an intervention, and this study showed that a social camp for burn patients can promote resilience. This study has presented a variety of factors that inhibit or encourage resilience and growth, such as demographic, individual, and social factors. We also present suggestions on interventions that may be used to promote growth following this adverse event, such as improving social support, coping styles and deliberate positive introspection.

## 1. Introduction

Improved care and treatments for burn injury have increased survival rates, but have led burn survivors to contend with greater long-term psychosocial and physical consequences [1]. It is recognized that burn injuries are traumatic experiences that can detrimentally impact an individual’s psychological and emotional wellbeing. Posttraumatic stress disorder occurs in 8 to 30% of the adult burn population [2], and 11 to 13% of child burn populations [3], and routine clinical practice accepts the absence of a mental health disorder as an acceptable goal. However, research has shown that postburn hospital admission rates for mental health conditions were 3.52–6.79 times as high compared to a matched uninjured cohort, suggesting that the absence of a diagnosed mental health is not adequate for optimal mental health recovery [4]. Despite this, some survivors adapt to psychosocial challenges better than others despite similar type, severity, bodily location, and physical consequences of the burn.

Resilience and posttraumatic growth (PTG) are related constructs but are not synonymous. Historically, PTG was seen to be part of resilience and they are often confused in the general post-trauma literature [5]. Resilience has been defined as the ability to maintain relatively stable, healthy levels of psychological and physical functioning, [6] and is about adapting or ‘bouncing back’ to the pre-trauma state. PTG has been defined as ‘the subjective experience of positive psychological change reported by an individual as a result of the struggle with trauma’ and describes development that has occurred beyond pre-trauma psychological functioning [7,8]. Resilience is static because it involves little or no change to an individual’s worldview, the event is already understandable, which allows a focus on the future. PTG, on the other hand, is dynamic because it involves a changing worldview, and it arises from deliberate rumination that focuses on the event with the purpose of making some sense of what happened [9,10].

Within the field of medical trauma, studies have investigated the barriers and enablers of resilience and PTG in cancer [11], serious paediatric illness [12], and spinal cord injury [13]. Across the different medical issues, resilience and PTG are associated with individuals being able to talk about their experiences, younger age, perceptions about the medical issue, and social support [11,12,13]. Specifically in burn injuries, there have been studies on the factors associated with resilience and PTG, and one review has investigated the correlates of PTG in adult burn survivors [9]. This review highlighted key areas that enabled PTG in this population (including function, quality of life, social support and optimism, hope, and new opportunities), which could be targeted following a burn injury to facilitate PTG. Though this review showcased important factors involved in PTG in burn patients, resilience, which is a separate though related construct, was not investigated. Further, there have been no attempts to synthesise the literature though, across ages and within paediatric populations. It is important to ascertain whether there are different factors that contribute to PTG in childhood and adolescence. 

This review aimed to assess the published literature that has been conducted in the field of resilience and PTG after burn injury. Specifically, the aim was to describe the constructs of resiliency and growth within the burn injury context, examine the risk factors that inhibit resilience or growth after burn (barriers), the factors that promote resilience or PTG after burn (enablers), and finally to assess the impact of interventions that have been tested that may facilitate resilience or growth after burn.

## 2. Method

This review was performed according to the recently updated Preferred Reporting Items for Systematic Reviews and Meta-Analyses (PRISMA) Guidelines [14,15]. The review was registered with the International Prospective Register of Systematic Reviews (PROSPERO ID295835).

### 2.1. Research Questions

1. What research has been conducted in the field of resilience and PTG after burn injury that examines the risk factors that inhibit resilience or growth after burn (barriers), the factors that promote resilience, or PTG after burn (enablers)?
2. What interventions have been tested that may facilitate resilience or PTG after burn?

### 2.2. Search Strategy

An electronic search was conducted in November 2021 on the databases PubMed, Medline (1966–present), Embase (1974–present), and PsycINFO for English-language peer-reviewed academic articles. No time restrictions were placed. Medical Subject Headings (MeSH terms) or equivalent terms were used for searching in Title, Abstract, and Keywords according to the requirements of the database. The search terms were broad to capture all relevant articles. MeSH or Indexed terms related to burn injury were combined using the Boolean operator “OR”. Separately, MeSH or Indexed terms related to burn survivorship, resilience, and posttraumatic growth were also combined using the Boolean operator “OR”. These two searches were combined with the Boolean operator “AND”.

Indexed terms: ((burn.mp. or Burns/) AND ((posttraumatic growth.mp. or exp Posttraumatic Growth, Psychological/) OR (Adaptation, Psychological/ or Resilience, Psychological/ or resilience.mp.))).

MeSH terms: (Burns) AND (Survivors/psychology) OR (Trauma and Stressor Related Disorders/psychology OR Trauma and Stressor Related Disorders/rehabilitation) AND (Resilience, Psychological OR Posttraumatic Growth, Psychological).

### 2.3. Inclusion and Exclusion Criteria

For question one (the investigation of barriers and enablers on resilience or PTG), qualitative or quantitative articles were included if they described factors that negatively or positively influenced resilience or growth. Participants could be adult or pediatric. Reviews were handled by extracting the relevant reference articles for inclusion in this review.

For question two, inclusion criteria were (1) adults and children with burn injury, (2) a psychosocial or physical intervention aimed at improving resilience or posttraumatic growth (this could be a psychotherapy (e.g., cognitive behavioral therapy), counselling, a psychoeducational strategy, peer support, or a physical or social activity, (3) any comparators, (4) outcomes involving resilience or posttraumatic growth, (5) all RCTs, and quasi-experimental intervention research studies. No time limits were set. Case reports, letters to the editor, conference abstracts, and grey literature were excluded. Articles published in languages other than English were excluded. 

**Figure 1:**
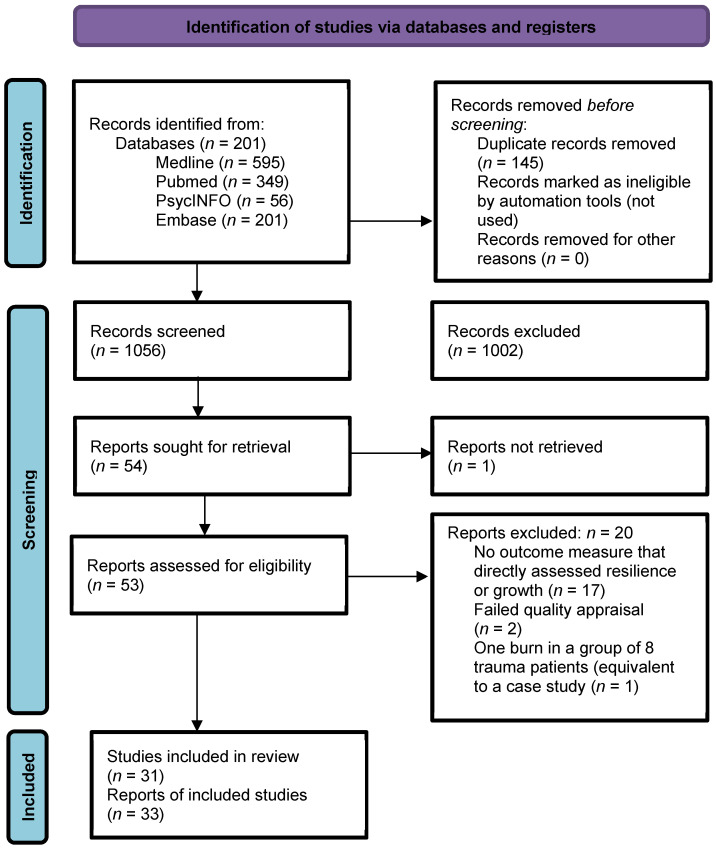
PRISMA diagram.

Titles and abstracts identified through the electronic search were reviewed independently by two authors (AW and LM) for inclusion. Each full-text review was completed by two authors to decide on eligibility for inclusion in the review. Any discrepancies in opinion were discussed between the authors. See the PRISMA diagram in Figure 1 for details.

### 2.4. Critical Appraisal

Quality assessment and risk of bias was assessed with the Joanna Briggs Institute critical appraisal tools [16]. The appropriate tool was selected depending on study design (see Table 1). Each study was assessed using these tools, the results were reviewed by the authors, and any discrepancies discussed. Two studies were excluded after quality assessment.

### 2.5. Data Extraction and Synthesis

The following information was extracted from the articles: authors, year and country, aim, study design, sample size, participant characteristics, clinical characteristics, outcome measures, statistical analyses, and findings.

**Table 1:** Quality Appraisal of Studies.

Study Type	JBI Quality Appraisal										
Cohort Studies	Were the two groups similar and recruited from the same population?	Were the exposures measured similarly to assign people to exposed unexposed groups?	Was the exposure measured in a valid and reliable way?	Were confounding factors identified?	Were strategies to deal with confounding factors stated?	Were the participants free of the outcome at the start of the study (or moment of exposure)?	Were the outcomes measured in a valid and reliable way?	Was the follow up time reported and sufficiently long for outcomes to occur?	Was follow up complete, were the reasons to loss to follow up described/ explored?	Were strategies to address incomplete follow up utilized?	Was appropriate statistical analysis used?
Martin 2021 [17]	NA	NA	Y	Y	Y	Y	Y	Y	Y	NA	Y
Martin 2017b [18]	NA	NA	Y	Y	Y	Y	Y	Y	Y	NA	Y
Analytical Cross-sectional Studies	Were the criteria for inclusion in the sample clearly defined?	Were study subjects and the setting described in detail?	Was the exposure measured in a valid and reliable way?	Were objective, standard criteria used to measure the condition?	Were confounding factors identified?	Were strategies to deal with confounding factors stated?	Were the outcomes measured in a valid and reliable way?	Was appropriate statistical analysis used?			
Ajoudani 2019 [19]	Y	Y	Y	Y	Y	Y	Y	Y			
Baillie 2014 [20]	Y	Y	Y	Y	Y	Y	Y	Y			
Bibi 2018 [21]	Y	Y	Y	Y	Y	Y	Y	Y			
Chen 2020 [22]	Y	Y	Y	Y	Y	Y	Y	Y			
He 2013 [23]	Y	Y	Y	Y	N	NA	Y	Y			
Holaday 1994 [24]	Y	Y	N	N	N	NA	Y	N	EXCLUDE		
Hwang 2020 [25]	Y	Y	Y	Y	Y	Y	Y	Y			
Jang 2017 [26]	Y	Y	Y	Y	Y	Y	Y	Y			
Jibeen 2018 [27]	Y	Y	Y	Y	Y	Y	Y	Y			
Masood 2016 [28]	Y	Y	Y	Y	N	NA	Y	U			
Quezada 2015 [29]	Y	Y	Y	Y	N	NA	Y	Y			
Rosenbach 2008 [30]	Y	Y	Y	Y	Y	Y	Y	Y			
Royse 2017 [31]	Y	Y	N	Y	Y	N	Y	Y			
Waqas 2016 [32]	Y	Y	Y	Y	N	NA	Y	Y			
Xia 2014 [33]	Y	Y	Y	Y	Y	N	Y	Y			
Yang 2014 [34]	Y	Y	Y	Y	Y	N	Y	Y			
Qualitative Research	Is there congruity between the stated philosophical perspective and the research methodology?	Is there congruity between the research methodology and the research question or objectives?	Is there congruity between the research methodology and the methods used to collect data?	Is there congruity between the research methodology and the representation and analysis of data?	Is there congruity between the research methodology and the interpretation of results?	Is there a statement locating the researcher culturally or theoretically?	Is the influence of the researcher on the research, and vice- versa, addressed?	Are participants, and their voices, adequately represented?	Is the research ethical according to current criteria, and is there evidence of ethical approval by an appropriate body?	Do the conclusions drawn in the research report flow from the analysis, or interpretation, of the data?	
Abrams 2018 [35]	Y	Y	Y	Y	Y	Y	N	Y	Y	Y	
Badger 2010 [36]	Y	Y	Y	Y	Y	Y	Y	Y	Y	Y	
Garbett 2017 [37]	Y	Y	Y	Y	Y	Y	Y	Y	Y	Y	
Habib 2021 [38]	Y	Y	Y	Y	Y	Y	U	Y	Y	Y	
Han 2020 [39]	Y	Y	Y	Y	Y	Y	N	Y	Y	Y	
Hunter 2013 [40]	Y	Y	Y	Y	Y	Y	Y	Y	Y	Y	
Kool 2017 [41]	U	U	U	U	U	Y	N	Y	Y	Y	EXCLUDE
Kornhaber 2014 [42]	Y	Y	Y	Y	Y	Y	U	Y	Y	Y	
Lau 2011 [43]	Y	Y	Y	Y	Y	Y	Y	Y	Y	Y	
Martin 2016 [44]	Y	Y	Y	Y	Y	Y	N	Y	Y	Y	
Martin 2017 [45]	Y	Y	Y	Y	Y	Y	N	Y	Y	Y	
McGarry 2014 [46]	Y	Y	Y	Y	Y	Y	U	Y	Y	Y	
McLean 2015 [47]	Y	Y	Y	Y	Y	Y	N	Y	Y	U	
Moi 2008 [48]	Y	Y	Y	Y	Y	Y	Y	Y	Y	Y	
Neil 2021 [49]	Y	Y	Y	Y	Y	Y	U	U	Y	Y	
Williams 2003 [50]	Y	Y	Y	Y	Y	Y	N	Y	N	Y	
Zhai 2010 [51]	Y	Y	Y	Y	Y	Y	N	Y	Y	Y	

Y = Yes; N = No; U = Unclear; NA = Not Applicable.

## 3. Results

### 3.1. Study Selection and Characteristics

This review included 33 studies. Study characteristics are outlined in Table 2 and Table 3. The studies comprised of two cohort studies, 15 analytical cross-sectional studies, 15 qualitative studies, and one qualitative intervention study. The settings of the studies included Australia (*n* = 8), China (*n* = 5), United States (*n* = 4), Pakistan (*n* = 3), Germany (*n* = 2), Korea (*n* = 2), United Kingdom (*n* = 2), Canada (*n* = 1), Iran (*n* = 1), Mexico (*n* = 1), Norway (*n* = 1), Saudi Arabia (*n* = 1), South Africa (*n* = 1), and Taiwan (*n* = 1). Thirty studies investigated adult populations, while only three investigated children or young people. In total, there were 1972 participants in the quantitative studies, and 205 participants in the qualitative studies. Of the quantitative studies, nine investigated resilience and eight investigated PTG. Four evaluated resilience with the Connor Davidson Resilience Scale, two used the Resilience Scale developed by Wagnild and Young [52], one used the Ego Resilience Scale developed by Block and Kremen [53], one used the State-Trait Resiliency Scale [54] and one used resilience scales developed for Mexican populations. Seven studies evaluated PTG with the Posttraumatic Growth Inventory [55,56], and one used the Perceived Benefit Scale [57]. For resilience, as measured by the CD-RISC, mean scores varied from 49.89 to 67.34 (Table 2). For PTG, as measured by the PTGI, mean scores (out of 5) varied from 1.26 to 3.18 [30], with mean scores over 2.5 recommended to represent a useful level of PTG [44]. Forty different outcome measures were used in the analyses, and this prevented the ability to quantitatively synthesize the data (Table 2). The qualitative studies were mixed, those that evaluated resilience stated this outcome, and those that evaluated growth reported the positive changes described by burn survivors.

### 3.2. Question One: Barriers and Enablers to Resilience or PTG

The results of the quantitative studies suggested that there are several barriers and enablers to resilience or PTG following a burn (see Table 2). Total body surface area of the burn was reported to be positively associated with PTG in four studies [18,19,20,31], although another study reported no association [30]. The relationship with stress differed between resilience and PTG, and was reported to differ between males and females. In terms of resilience, three studies reported that increased stress [22,28] or subclinical symptoms of post-traumatic stress disorder [21] hindered resilience following a burn. Bibi et al., [21] reported that this barrier to resilience was further associated with gender, as females reported higher traumatic stress and lower resilience. Masood et al. also reported similar findings about gender, resilience, and distress [28]. However, Yang et al. [34] reported resilience to be higher in females. For PTG, stress and PTG co-exist, stress is reported to precede growth, and has a positive association with growth [18,20,30], with females reporting more growth than males [30]. In terms of barriers to PTG, time postburn was identified as a factor, with higher risk for poor PTG occurring in the year following a burn [32]. Younger age was associated with resilience [29], but not PTG [30], and other studies found no association between age and either construct [23].

Good social support was a strong enabler of both resilience and PTG [18,20,25,30]. Spirituality had a positive effect on both resilience and PTG [17,22,23], Additionally, some studies found personality factors like optimism promoted resilience [32], extraversion promoted PTG, and neuroticism was a barrier to PTG [27]. Other social factors were influential, such as marital status (divorced people being more resilient) and occupation (farmers and workers being more resilient) [23]. PTG was found to be influenced by returning to work [18], narrative restructuring to reframe the accident [20], and coping mechanisms [20,28].

In terms of the qualitative studies, both barriers and enablers to resilience were identified (see Table 3). Barriers included seeing others in distress [58], needing to be prepared to ask for help [58], maladaptive or negative coping styles [45], and worries about stigma or rejection [45]. Another common barrier found by several studies was self-consciousness or worries about the reactions and perceptions of others [17,36,45]. Barriers to PTG were assessed by Martin et al. [45], and factors included emotional barriers (e.g., fear of rejection, self-consciousness, embarrassment), situations barriers (e.g., questions from others), and behavioral barriers (reactions from others, pressure garments), as well as avoidant coping styles. Enablers of resilience included skills-based factors such as utilizing problem-solving skills [35], improving social competence [35,37], having a sense of autonomy [35], resourcefulness [35], critical thinking [35], and detaching from negativity [35]. Other themes included factors related to personality such as having empathy [35,37], having strong willpower [35,39], having a sense of optimism or hope [17,35,39], using humor [35,40], and being curious about the world [39]. Positive coping was also indicative of enhanced resilience [35,37,39,40,42,47,51], and shifting self-perception [37,49] and sharing or expressing one’s feelings [37]. Having a sense of altruism and spirituality were also identified as enabling resilience [17,35,39,40,44,46,51], as was not being ashamed or embarrassed about the scar [40]. The two most common factors enabling resilience were having a positive life purpose or meaning [35,37,39,40,42,44,47,51], and having good social or peer support [17,37,39,40,44,49,51].

Four qualitative studies looked at barriers and enablers of PTG [37,40,44,51]. Again, across all studies, positive relationships were important in fostering PTG [37,40,44,51]. Further, shifting self-perception and life outlook [37,51], acknowledging one’s personal strength [44,51], new possibilities [44], spirituality [24], gratitude [40,44], humor [40], managing emotions [51], effective coping [40,51], altruism [51], and more sharing with others [51].

### 3.3. Question Two: Interventions Targeting Resilience or PTG after Burns

There was only one qualitative study that investigated the impact of a burn camp for children on psychosocial outcomes and found that the social environment of a burn camp greatly enhanced resilience [49]. In particular, the camp improved the children’s confidence, psychological recovery, it normalized their experiences, and provided social support.

## 4. Discussion

This study aimed to describe the constructs of resilience and growth within the burn injury context, examine the risk factors that inhibit resilience or growth after burn (barriers), the factors that promote resilience or PTG after burn (enablers), and to assess the impact of interventions that have been tested that may facilitate resilience or growth after burn. Findings were mixed for most studies; however, there were factors related to demographic information, burn-specific characteristics, person-specific factors, psychopathology, and psychosocial and social factors that were evidenced to be related to resilience and PTG. It is important to remember the differences in the construct of resilience and growth.

For age, one study found that being younger promoted resilience [29], another that showed older participants demonstrated more PTG [30], and another that contradicted this finding of no significant association between age and PTG [23]. In other populations, PTG is typically associated with younger age [59,60], and thus these results should be interpreted with caution given the variability in findings. For gender, associations with resilience were reported to be higher in women in one study [28], yet lower in others [21], [28]. In these latter two studies, lower levels of resilience presented with higher levels of stress symptoms. In addition, the gender differences were thought to be mediated by higher levels of social support for males in the local cultural environment, but this was not statistically investigated. However, associations between gender and PTG differed to the associations between gender and resilience, with women reporting higher levels of PTG compared to men [30], although another study found no gender differences [23].

Most studies showed larger TBSA being associated with higher levels of PTG [18,19,20,23,31]; however, another study found no relationship between TBSA and PTG [30], but this was possibly due to study design. The positive association between TBSA and PTG might be due to the influence of high levels of stress leading to more growth [20], which is consistent with theories of PTG [8,61]. In addition, scar visibility might affect resilience or PTG [9,40,43], although functionality might be more important to recovery than aesthetics [40]. As time moves on after the burn event, burn survivors may do better [57], and those burnt as children might do better in terms of social support compared to adults [43]. Not all studies reported time since burn, and further research is required to assess long-term trajectories.

Individual factors related to resilience or PTG included personality and potential psychopathology. Optimism is a personality factor that might contribute to resilience [21,23] and has been suggested to boost PTG by affecting subjective wellbeing [23]. Optimism has been found to promote PTG in other clinical populations such as patients with HIV [62]. The personality trait of extraversion was found to predict positive change, whilst neuroticism was found to increase distress and impede PTG [27]. 

The role of spirituality is interesting, those who have a faith find more inner strength, and both spiritual change and inner strength are components of PTG [55]. Spirituality has been shown to have a positive association with resilience [31] and growth [19,27] and the importance of offering pastoral support to patients should not be underestimated [17].

Adaptive coping mechanisms (such as positive reframing, humor, planning, resourcefulness, downward comparison, acceptance, and focusing on the future in a positive way) were all found to promote resilience and PTG [18,26,35,39,40,43,44,48,58]. Distress was found to be related to resilience and PTG, and it is thought that this is because those in distress might need to adopt new ways of thinking about a situation that is not able to be changed. Stress is thought to precede PTG [18,20] and stress and PTG co-exist [30]. This theory is supported in research with other populations, whereby more stress or distress is associated with more PTG [12,63]. Depression and anxiety were found to impede resilience and PTG in some studies [26,36,40], but not others [18]. Studies in other populations (i.e., cancer) have found that PTG is related to fewer symptoms of depression [64]. Burn-related studies that found depression to be a barrier to growth, suggest that this is due to the overwhelming of coping resources that are necessary for growth to occur [58] or due to the negative reframing that naturally occurs in depression [18].

Social support was overwhelmingly identified in this review to facilitate both resilience and PTG. Specifically, we found that social support [17,18,20,22,31,34,37,39,40,44,49,51,65], spirituality/religion [17,31,35,39,40,44,46,51], and a positive life purpose [35,37,39,40,44] were the most commonly reported enablers of resilience and PTG. Further, having quality relationships [26] and recognizing you are not alone [17,31] were notably important. It should be stated though, that cultural differences may impact on the relationship between spirituality and religion [51]. Concerns about burdening others by sharing experiences were a social barrier that was a barrier to PTG [40,44]. The results found in this study are in line with research on PTG in non-burn populations, such as rheumatoid arthritis [59]. The process by which growth occurs can only be explored with rich contextual data from qualitative studies, and these suggest that growth arises from deep introspection [39] that leads to a new worldview to create coherence in their own personal narratives of their lives [48] and the need to find some meaning in the situation [51]. This is similar to PTG after other types of trauma, and is central to related background theories [55,65].

One qualitative study conducted an intervention to promote resilience and found that encouraging social support via a burn camp could help improve resilience in children [49]. One other study audited peer support as a potential mechanism for intervention to promote resilience and PTG and noted it would be a promising area to target [58], which is logical given the overwhelming evidence in this study supporting social factors in the encouragement of resilience and PTG [17,18,20,22,31,34,37,39,40,44,49,51,65]. Future studies should target adult populations, as there were no interventions for this group despite poor psychosocial outcomes being an issue for this population [4]. Interventions could focus on methods to promote deliberate rumination and introspection [39], teach adaptive coping styles [58], and teach clinicians and parents how to recognize ‘red flags’ and promote ‘green flags’ (i.e., symptoms of PTSD and PTG) [58]. Finally, as it appears that depression is a factor in resilience and PTG, clinicians should screen burn patients for symptoms of depression, to optimize psychosocial recovery and ensure a good personal environment for PTG to occur.

### Interventions, Limitations, and Future Considerations

This study found several limitations in the existing literature on resilience and PTG in populations that have experienced a burn, which could drive future research and clinical practice. Firstly, the studies were heterogeneous in their scope and methodology, which made synthesis difficult and rendering us unable to conduct a meta-analysis. There needs to be more research in the underlying mechanisms of both resilience and PTG. Further to this, there were only two small longitudinal studies on the progression on PTG [18,58], which makes understanding PTG as a process difficult. There also needs to be more research conducted on child and adolescent populations, given this is a large demographic for burn injuries. We do not currently know how the presentation or trajectory of PTG differs in pediatric versus adult populations.

## 5. Conclusions

Resilience and PTG are important constructs to understand given that individuals who experience a burn injury are a high-risk population for longer term mental health issues. This study has presented a variety of factors that inhibit or encourage resilience and PTG, such as demographic, individual, and social factors. We also present suggestions on interventions that may be used to promote growth following this adverse event, such as improving social support, coping styles, and deliberate positive introspection. Ideally, clinicians and family members/parents would also be aware of the importance of resilience and PTG and be able to look out for and promote these phenomena when treating burns patients.

## Figures and Tables

**Table 2:** Quantitative Study Characteristics.

First Author, Year and Country	Aim	Study Design	Study Population (*n*, Age, Gender %)	Burn Characteristics (TBSA %, Time since Burn)	Outcome Measures	Statistical Analyses	Findings	Interpretation
Ajoudani, F.; Jafarizadeh, H.; Kazamzadeh, J. 2019 [19] Iran	To investigate the relationship between social support and posttraumatic growth (PTG) in Iranian burn survivors, as mediated by their perceptions of spiritual well-being.	Analytical cross-sectional	*n* = 102 Male %: 40 Age $\bar{x}$ (*SD*): 27.5 (8.14)	TBSA: mean (*SD*): 32.9 (6.1) Time since burn: Not reported (Study inclusion criteria >1year post burn).	Posttraumatic Growth Inventory (PTGI), Spiritual Well-Being Scale, the Multidimensional Scale of Perceived Social Support (MSPSS)	Descriptive statistics, univariate analyses, Pearson’s correlation test, Anderson and Gerbing’s two-step modelling procedure, chi-square test, comparative fit index, root mean square error of approximation, Tucker-Lewis fit index, standardised root mean residual	PTGI $\;\bar{x}$ 78.13 (range 0–105). Social support $\bar{x}$ 56.96 (range 12–84). Spirituality $\bar{x}$ 92.15 (range 20–120). Positive correlation between PTGI and TBSA (r = 0.44, *p* = 0.01). PTGI scores different by education level category (F = 3.02; *p* = 0.03). Positive correlation between PTGI and social support (r = 0.332, *p* < 0.01) and spirituality (r = 0.371, *p* < 0.01). Positive correlation between social support and spirituality (r = 0.442, *p* < 0.01). The effect of social support on PTG decreased when spirituality included in model (b = 0.21, *p* < 0.001)	Burn survivors who perceived a higher level of social support experienced greater PTG. It was proposed that perceived social support is a key element for the psychological adjustment of burn survivors. The mediating role of the spirituality suggests that social support increases PTG, both directly and indirectly. There is a positive association between TBSA scores and PTGI scores. Improving social support and spiritual wellbeing might be an effective strategy for enhancing PTG among burn survivors.
Baillie, S.E. Sellwood, W. Wisely, J.A. 2014 [20] UK	To examine PTG using quantitative measures of growth, social support, coping styles and dispositional optimism to determine the potential predictors of PTG. To assess quality of life, and to clarify the relationship between PTG and distress.	Analytical cross-sectional	*n* = 74 Male %: 42 Age mean (*SD*): 45.7 (17.11)	TBSA mean (*SD*): 9.4 (*SD* not reported) Time since burn mean: 69 weeks (range 4–624 weeks)	PTGI, Coping with Burns Questionnaire (CBQ), MSPSS, The Impact of Event Scale-Revised, BSHS-B40, Life Orientation Test-Revised (LOT-R)	Descriptive statistics, t-tests, one-way ANOVA, correlations, hierarchical linear (stepwise) regression analysis, scatter plots.	PTGI and TBSA were positively correlated (r = 0.47, *p* < 0.01). PTGI scores and time since burn positively correlated (r = 0.34, *p* < 0.01). Burns involving both hands and face $\bar{x}$ = 2.86, (95%CI 2.13, 3.79) reported more growth than burns to the body $\bar{x}$ 1.01 (95%CI 0.70, 1.39) or face only $\bar{x}$ 1.15 (95% CI 0.45, 2.20). Positive correlation for PTG scores with PTS (r = 0.32, *p* < 0.01) and PTG scores with social support (r = 0.22, *p* < 0.05). Avoidance coping associated with PTG (r = 0.43, *p* < 0.01). PTG scores higher with more avoidance coping (b = 0.581, *p* = 0.001). greater TBSA (b = 0.132, *p* = 0,002) more instrumental/action coping (b = 0.495, *p* = 0.005), more social support (b = 0.407, *p* = 0.005).	The process of growth emerges from distress, aided by coping styles and social support. Burns involving the face and hands reported more growth The more severe the burn the more growth experienced. More PTG with more time postburn. Facilitating growth through narrative may be beneficial. Patients could also be assisted to establish or renew meaningful social support networks.
Bibi, A; Kalim, S; Khalid, M.A. 2018 [21] Germany	To investigate the relationship between posttraumatic stress disorder (PTSD) symptoms and resilience among burn patients in Pakistan and exploring the influence of gender.	Analytical cross-sectional	*n* = 70 Male %: 49 Age median (IQR): 27.5 (IQR 13)	TBSA categorsied. Range 10–30%. Time since burn not reported.	PTSD CheckList-Civilian Version (PCL-C), Connor-Davidson Resilience Scale (CD-RISC)	Descriptive statistics, Spearman’s Rank-Order correlation, ANCOVA	A strong negative correlation between PTSD and resilience among burn patients (r = −0.72, *p* < 0.001). A significant effect of gender on PTSD among burn patients F (1, 64) = 14.22, *p* < 0.001; η2 = 0.18). A significant effect of gender on resilience among burn patients (F (1, 64) = 22.03, *p* < 0.001 (η2 = 0.25)). Females had generally lower resilience than males.	Low levels of resilience are associated with higher symptoms of PTSD. Females had more severe PTSD symptoms and lower resilience than males; likely due to cultural factors and differing peer supports for men and women in Pakistan. Culture-based rehabilitation strategies should be planned Improving optimism and faith could also help.
Chen, Y.; Lu, M.; Weng, L.; Huang, P.; Wang, C.; Pan, H. 2020 [22] Taiwan	To explore the relevant factors affecting resilience in burn patients who had experienced the Formosa Fun Coast Explosion.	Analytical cross-sectional	*n* = 30 Male %: 63 Age mean (*SD*): 22.8 (4.30)	TBSA mean (*SD*): 45 (16.4) Time since burn not reported (Study inclusion criteria was 3–5 months post burn).	Resilience Scale, Perceived Stress Scale	Descriptive statistics, Kolmogorov-Smirnox test, Pearson correlation, t-test, one-way ANOVA, Scheffe’s post hoc test, Kendall’s tau coefficient, Mann-Whitney U test, Kruskal-Wallis test, multivariate linear regression.	Resilience score $\bar{x}$ 132.7 (moderate). Stress $\bar{x}$ 25.4. Every 1-point increase in stress level decreased resilience by 1.69 points in the stepwise regression.	Perceived stress was the key predictor of resilience: The higher the level of stress, the lower the resilience in participants. Screening recommended to determine stress levels for targeted intervention.
He, F.; Cao, R.; Feng, Z’.; Guan, M.; Peng, J. 2013 [23] China	Investigation into the effects of dispositional optimism and psychological resilience on the subjective wellbeing of burn patients.	Analytical cross-sectional	*n* = 410 Male %: 75 Age mean (*SD*): 25.2 (2.76)	TBSA not reported (Study inclusion criteria was 20–40% TBSA). Time since burn not reported.	LOT-R, CD-RISC, Subjective Wellbeing Scale	Anderson and Gerbing’s two-step modelling, chi-square, root mean square tests, Bootstrap estimation procedure, confirmatory factor analysis.	The effect of dispositional optimism on wellbeing through psychological resilience was 17.9%. Dispositional optimism and psychological resilience had a direct effect on subjective wellbeing. and an indirect effect on subjective wellbeing through psychological resilience.	Burn patients with high optimism are more likely to be capable of recovering from stressful situations. Dispositional optimism and psychological resilience act as protective factors, increasing the ability of burned patients to recover from their injury.
Hwang, S.; Lim, E.; 2020 [25] Korea	To identify the differences in the level of depressive symptoms, social support, and PTG among patients with severe burns by treatment phase and the factors associated with PTG in the acute and rehabilitation phases.	Analytical cross-sectional	*n* = 179 Male %: 78 Age mean (*SD*): 45.8 (12.89)	TBSA $\bar{x}$ (*SD*): 19.3 (17.17) Acute phase: 16.7 (14.63) Rehabilitation phase: 20.9 (18.44) Time since burn not reported for overall population, broken down into groups Acute phase: 64 days * Rehabilitation phase: 685 days *	Becks Depression Inventory II, Social Support Scale (SSS), PTGI, a general characteristic survey	Descriptive statistics, Chi-square test, t-tests, Pearson’s correlation coefficients, regression analysis.	Acute group: PTGI $\bar{x}$ 44.13 (SD 15.01). Depression $\bar{x}$ 15.93 (*SD* 9.68). Rehabilitation group: PTGI $\bar{x}$ 40.32 (*SD* 15.71). Depression $\bar{x}$ 20.55 (*SD* 13.31). PTGI and depression negatively correlated in both groups, Acute: r = −0.257 (*p* = 0.035) Rehabilitation: r = −0.378 (*p* < 0.001) PTGI and social support positively correlated for both groups. Acute: r = −0.4017 (*p* = 0.001) Rehabilitation: r = −0.510 (*p*< 0.001)	There is an inverse correlation between depression and PTG. There are differences in depressive symptoms in burn survivors’ dependent on their phase of treatment. The first-year post burn incident is when individuals are most susceptible to., depression. Burn patients management of depressive symptoms, in the acute and rehabilitation phase. Social support is protective Contemplative processes may lead to PTG.
Jang, M.; Park, J.; Chong, M.; Sok, S. 2017 [26] Korea	To examine and identify the factors influencing the degree of resilience among Korean burn patience.	Analytical cross-sectional	*n* = 138 Male %: 67 Age mean (*SD*): 46.8 (5.43)	TBSA mean not reported for overall sample. <29: 113 (81.8) 30–39: 7 (5.1) 40–49: 7 (5.1) 50–59: 5 (3.6) >60: 6 (4.4) Time since burn not reported.	Korean adaption of the Resilience Scale, Beck Depression Inventory, State Trait Anxiety Inventory, Self-Esteem Scale, Family Support Scale	Descriptive statistics, Pearson’s correlations, multiple regression analysis	Resilience $\bar{x}$ (*SD*): 86.15 (11.70). Positve correlation for resilience with self-esteem (r = 0.524, *p* < 0.001) and resilience with family support (r = 0.523, *p* < 0.001). Negative correlation for resilience with depression (*r* = −0.496, *p <* 0.001) and resilience with anxiety were negatively correlated (*r* = −0.541, *p <* 0.001) Self-esteem (β = 0.35, *p <* 0.001), and family support (β = 0.29, *p <* 0.001) predicted resilience.	Anxiety, self-esteem, family support, educational level, income, and family support affected resilience. Depression was a major contributor to poor self-perception and positive outlook.
Jibeen, T.; Mahfooz, H.; Fatima, S. 2018 [27] Saudi Arabia	To examine the associations between personality traits, spiritual transcendence, positive change, and psychological distress in a burn sample.	Analytical cross-sectional	*n* = 96 Male %: 71 Age mean (*SD*): 30.4 (13.08)	TBSA mean not reported, (Study inclusion criteria: 20–80%) Time since burn not reported.	NEO Five-Factor Inventory, Depression, Anxiety, Stress Scales-21 (DASS-21), Spiritual Transcendence Index, Perceived Benefits Scales	Correlations, stepwise regression analyses	Positive correlation for perceived benefits with extraversion, spirituality. Negative correlation of perceived benefits with distress and neuroticism (*p* values not stated). Positive change was predicted by longer LOS, less spirituality, more neuroticism, and less extraversion. Distress was predicted by longer LOS, less spirituality, more neuroticism, and less extraversion.	Spiritual transcendence in burn patients is likely to protect them from negative consequences of burn trauma and promote PTG, which may lead to successful adaptation. Neuroticism positively predicts psychological distress and negatively predicts positive change. Extraversion negatively predicts psychological distress and positively predicts positive change. Those with neurotic traits may be less likely to benefit from spiritual transcendence to reduce psychological distress, while those manifesting extrovert traits may be more likely to benefit.
Martin, L.; Byrnes, M.; McGarry, S.; Rea, S.; Wood, F. 2017b [18] Australia	To determine the nature of the relationship between growth, stress, and quality of life post burn (via the health-related quality of life outcome measures), and whether PTG changed over time in individuals who have sustained a burn.	Longitudinal cohort study	*n* = 73 Male %: 69 Age $\bar{x}$ (*SD*): 43.0 (14.00) Non-acute group: Age $\bar{x}$ 41.8 (14.5) Acute group: Age $\bar{x}$ 44.3 (13.5)	TBSA $\bar{x}$ (*SD*): 18.5 (20.10) Non-acute: $\bar{x}$ 32.7 (21.20) Acute group: $\bar{x}$ 6.1 (5.90) Time since burn Non-acute: >6 months Acute group: <6 months	SF-36 Quality of Life, BSHS-B40, PTGI, DASS-21	Longitudinal regression analysis, chi-square, Wilcoxon Rank Sum (Mann-Whitney) tests, multiple linear regression analysis, *t*-tests.	PTG did not differ between gender, age at injury, time since injury, marital status or Australian born. TBSA had a positive effect on total PTGI scores that was close to significance. DASS-21 curvilinearly associated with PTGI highly significant (b = 1.3, *p* < 0.0001, rho = 0.76). More growth was reported at moderate levels of total DASS-21 scores, with this reducing at higher levels of depression.	PTG associated with higher levels of stress. Less PTG occurs as mental health and mood improve. Depression is a barrier to growth. possibly inhibiting the ability to use helpful thinking styles, reducing motivation, and disrupting the capacity to cope. This urges the need for early identification, diagnosis, and treatment of depression. Growth scores are highest at moderate levels of recovery, then reduce again while recovery continues as burn survivors return to work and resume everyday life. Returning to work is significant in psychological recovery after burn.
Martin, L.; Rea, S.; Wood, F. 2021 [17] Australia	To assess the relationship between coping styles and PTG in an adult burn population.	Longitudinal cohort study	*n* = 36 Male %: 64 Age $\bar{x}$ (*SD*): 43.0 (15.26)	TBSA $\bar{x}$ (*SD*): 11.5 (11.35) Time since burn $\bar{x}$: 233 days	BriefCOPE, PTGI, DASS-21	Descriptive statistics, univariate regression analysis, multivariate regression analysis.	PTG is expected to increase by 2.0 units for every unit increase in acceptance, and by 1.7 for every unit increase in positive reframing, and by 2.3 for every unit increase in religious coping strategies. Depression is expected to increase by 1.3 units for every unit increase in behavioural disengagement, and by 0.7 for every unit increase in self-blame, and by 0.6 for every unit increase in venting.	Three “approach” coping strategies were predictors of PTG: positive reframing, acceptance and use of religion. “Avoidant” coping venting, self-blame, and behavioural disengagement were predictive of depression after burn. Coping mechanisms associated with depression can be used as ‘red flags’ for early depression screening. Coping mechanisms associated with PTG can be used in interventions (i.e., providing pastoral support to burn patients). Depression screening can indicate those in need of support.
Masood, A.; Masud, Y.; Mazahir, S. 2016 [28] Pakistan	To explore gender differences in resilience and psychological distress of patients with burns.	Analytical cross-sectional	*n* = 50 Male %: 50 Age categorised. Mean (*SD*): unclear. Range 16–48 years	TBSA not stated Time since burn not stated (criterion >6m postburn)	State-Trait Resilience Inventory; Psychological distress scale	Descriptive statistics, Pearson correlation, *t*-test, linear regression.	Mean resilience for males was higher than females for each of the four types of resilience (interstate, intrastate, intertrait, and intratrait). Mean levels of psychological distress were higher for females reaching statistical significance. Lower levels of intrastate, intertrait, and intratrait resilience and higher levels of interstate resilience predicted psychological distress,	Women show less resilience than men after trauma, and show more distress. This is interpreted from the following bservations: Inner strength (Intratrait) is higher in males than women. The positive relationship between interstate resilience and intrastate resilience in males is indicative of more social support. Males have wider social support networks than females in Pakistan and because of the social structure.
Quezada, L.; Gonzalez, M.T.; Mecott, G.A’. 2016 [29] Mexico	To explore the roles of both the patient’s and caregivers’ resilience and PTS in paediatric burn survivor adjustment.	Analytical cross-sectional	*n* = 51 Patients: Male *n* = 29 Age $\bar{x}$ (*SD*): 12.0 (3.00) Caregivers: Male *n* = 12 Age $\bar{x}$ (*SD*): Females: 36.9 (7.13) Males: 45.8 (8.9).	TBSA $\bar{x}$ (*SD*): 31 (19.00) Time since burn mean: 6 years (4.14)	Resilience Questionnaire for Children and Adolescents, Mexican Resilience Scale for Adults, Davidson Trauma Scale	Spearman’s correlation, structural equation modelling, Tucker-Lewis index, comparative fit index, root mean square error of approximation	Patients: Resilience scores $\bar{x}$ (*SD*) 128.3 (21.63) range 105 to157. Caregivers: Resilience scores $\bar{x}$ (*SD*) 141.43 (19.42) range 75 and 168. For patients: More resilience negatively correlated with age at burn r = −0,414, *p* > 0.001), was also a predictor of resilience. Higher levels of female caregiver avoidance and intrusion symptoms negatively impact child resilience (r = 0.389. *p* < 0.05).	Higher resilience in paediatric burn survivors associated with being younger at the time of the burn. Caregiver PTS intrusion symptoms were the second-best predictor of patient resilience. The higher the resilience in caregivers, the lower their avoidance symptoms, which further results in a lower severity of intrusion symptoms. Psychological responses of caregivers affect wellbeing and positive adjustment of patients; thus, psychological services for caregivers would likely have a dual benefit for both caregivers and patients.
Rosenbach, C. & Renneberg, B. 2008 [30] Germany	To investigate PTG in burn patients after discharge from the hospital for acute treatment, and to identify correlates facilitating or preventing the acceptance of positive change.	Analytical cross-sectional	*n* = 149 Male %: 57 Age mean (*SD*): 44.0 (14.40)	TBSA mean (*SD*): 32.2 (18.10) Time since burn mean: 4 years	PTGI, CBQ, Symptom Checklist, SSS, SF-12	Pearson’s correlations, multiple hierarchic linear regressions,	Active coping was the strongest predictor of PTG.. Women reported significantly higher levels of PTG than men. Older adults (53–88 years) reported the highest levels of PTG (M = 3.47, 44–52 years; M = 2.99, 37–43 years M = 3.21, 16–36 years, M = 3.09). Participants reported that they used more active problem-focused coping strategies than avoidant coping-strategies (*t =* 11.64, df = 147, *p <* 0.001). No gender differences regarding coping, social support, or emotional distress. Injury severity was not associated with PTG.	The PTG in the sample was pronounced regarding more Appreciation of Life, enhancement of Relationships with Others, and greater sense of Personal Strength. The use of active coping strategies and a higher level of perceived social support were found to be strongly associated with more PTG, with this being an intervention avenue that ought to be considered by treatment teams. Distress and growth can co-exist.
Royse, D. & Badger, K. 2017 [31] USA	To investigate the occurrence of near-death experiences in burn survivors and its possible effects on PTG and life satisfaction following injury.	Analytical cross-sectional	*n* = 92 Male %: 53 Age mean (*SD*): 47 years (*SD* not reported) range 21–80.	TBSA mean (*SD*): 46.0% (*SD* not reported) range 1–95%. Time since burn mean: 26.8 (*SD* not reported) Near death experience group: 13.3 (*SD* not reported) Non-near death experience group: 13.6 (*SD* not reported)	Near Death Experience Scale (NDES), SLS, Posttraumatic Growth Inventory- Short Form (PTGI-SF)	One-way ANOVA, t-tests	Burn survivors who indicated that their religion was not a source of strength and comfort to them had the lowest scores on the NDES (F = 3.1, df = 2.91, *p* = 0.05), and those that indicated that their religion was a source of strength and comfort to them (“a great deal” vs. “a little” or “none”) had the highest scores on life satisfaction (F = 5.97, df = 2,86, *p* = 0.004). PTGI-SF scores were positively correlated with TBSA (r = 0.24, *p* = 0.02).	No significant correlations between level of PTG and years since injury, age, or gender. Findings reflected a positive association of PTG with injury severity when the sample was divided to <30 or >30% TBSA. Religion/spiritually acts a protective factor and supports PTG. Helping professions should integrate the role of religion/spiritually into their interventions, and training around how to do so should be provided.
Waqas, A.; Nabveed, S.; Bhuiyan, M.M.; Usman, J.; Inam-al-Huq, A.; Cheema, S.S. 2016 [32] Pakistan	To investigate and compare ego resiliency levels and the degree of social support in patients with a burn injury and their healthy counterparts.	Analytical cross-sectional	*n* = 160 burn *n* = 80 control *n* = 80 Male %: 24 Age mean (*SD*): 34.9 (11.20)	TBSA mean not reported. Time since burn mean (*SD*): 6.3 (4.70)	Urdu version of the Ego Resilience Scale, MSPSS	Descriptive statistics, chi-square test, t-test, point biserial correlations	Ego resiliency score $\bar{x}$ (SD): 2.82 (.63). There were no significant differences in mean scores on ego resiliency scale between the burn patients and their healthy counterparts. Patients with a burn injury were associated with lower scores on the MSPSS (r = 0.455, *p* < 0.001). They reported lower scores on social support from their significant other and family and friends in comparison to their healthy counterparts.	Lack of social support among burn patients can negatively influence their survival, physical and mental health. The care of burn patients should involve families, significant others, and friends.. Resources should educate social supports on the physical and mental health effects of burn injuries as this could improve the clinical outcome of burn patients.
Xia., Z.; Kong, Y.; Yin, T.; Sji, S.; Huangm, R.; Cheng, Y. 2014 [33] China	To investigate the impact of acceptance of disability and psychological resilience on PTSD in patients with burns.	Analytical cross-sectional	*n* = 127 Male %: 67 Age mean (*SD*): Overall age not reported. Age categorised. Range 18–60	TBSA mean not reported. (Study inclusion criteria: second-degree burns with tbsa >10% or third-degree burns with tbsa >5%). Time since burn not reported.	PCL-C, Acceptance of Disability Scale, CD-RISC	T-tests, ANOVA, descriptive statistics, Pearson’s correlations, multiple regression analysis	Resilience was negatively correlated with re-experiencing (r = −0.251, *p* < 0.001), avoidance/numbing (r = −0.316, *p* < 0.001), hyperarousal (r = −0.212, *p* < 0.001), and total PTSD scores (r = −0.308, *p* < 0.001). Lack of self-improvement was a predictor for PTSD (*p* = 0.002).	Trauma negatively affects resilience, and resilience is required for PTSD prevention. Lack of self-improvement was a predictor for PTSD.
Yang, Z.; Wang, J.; Zhang, B.; Zeng, Y.; Ms, H. 2014 [34] China	To investigate factors that influence resilience in patients with burns during rehabilitation, and to provide theoretical guidance for psychological crisis prevention and intervention.	Analytical cross-sectional	*n* = 129 Male %: 81 Age $\bar{x}$ (*SD*): 34.2 (10.22)	TBSA mean (SD): not reported, Categorised into mild (*n* = 53), moderate (*n* = 55), severe (*n* = 21). Time since burn mean: not reported	CD-RISC, SSS, Simplified Coping Styles Questionnaire	Descriptive statistics, *t*-tests, ANOVAs, rank sum test, Pearson’s correlation, linear regression analysis, multivariate regression analysis	Patients with severe burns: Higher resilience (F = 3.10, *p* = 0.049) Higher tenacity (F = 3.48, *p* = 0.034) Higher strength (F = 3.64, *p* = 0.029) Females—more resilience and optimism Divorcees—more strength Age, income and education—not difference in resilience. Optimism and social support correlated (r = 0.295, *p* < 0.01).	The level of resilience in females. Social support may enable patients to have an optimistic attitude during treatment, supporting resilience. Psychological intervention should include guidance with coping strategies. This should include helping to change the individual’s environment, encouraging active acceptance of lifedtyle changes, and helping them to improve their resilience.

Note: * Where “average” has been stated in the manuscript, mean has been assumed. Abbreviations: BRS-CSSEI: Burn Related Supplement to the Copper-Smith Self Esteem Inventory; BSHS-B40: Brief Version of the Burn Specific Health Scale; CBQ: Coping with Burns Scale; CD-RISC: Connor-Davidson Resilience Scale; CSSEI-C: Copper-Smith Self Esteem Inventory, Form C; DASS21: Depression Anxiety Stress Scales; LOT-R: Life Orientation Test-Revised; MSPSS: Multidimensional Scale of Perceived Social Support; NDES: Near Death Experience Scale; PCL-C: PTSD Checklist-Civilian Version; PTGI: Posttraumatic Growth Inventory; PTGI-SF: Posttraumatic Growth Inventory-Short-Form; PVPSS: Perceived Value of Peer Support Scale; SLS: Satisfaction with Life Scale; SSS: Social Support Scale.

**Table 3:** Qualitative Study Characteristics.

Authors, Year, and Country	Aim	Sample Size	Participant Age	Study Design	Age at Time of Burn	TBSA	Time since Burn	Intervention	Results
Abrams, T.E.; Ratnapradipa, D.; Tillewein, H.; Lloyd, A.A. 2018 [35] USA	To investigate the holistic health of adult patients who had sustained major burn injuries.	*n* = 8 (7 male)	Mean 54.38 years Range 18–65 years	Heuristic phenomenological study	Mean 42.38 years	Range 20–98%	Mean 9.3 years	Not applicable	Four themes (1) Problem-solving skills (2) Social competence (3) life purpose (4) autonomy
Badger, K. & Royse, D. 2010 [36] USA	To explore perceptions of peer support in recovery.	*n* = 30 (19 male)	Mean = 41 (*SD* = 10.9) Range 19–71 years	Kvale’s (1996) model for a qualitative interview investigation	Not reported	Mean 60% (*SD* 20.76) range 25–93%	Mean = 14 years (*SD* = 13)	Peer support	Six themes (1) Positive regard for peer support (2) provision of hope and perspective (3) experience of belonging and affiliation (4) emotional cost (5) helping other’s helps oneself (6) mental preparedness to reach out
Garbett, K.; Harcourt, D. & Buchanan, H. 2017 [37] United Kingdom	To build on current research by qualitatively exploring the positive outcomes that may be present following a burn.	*n* = 10	Not reported	Thematic analysis of longitudinal blog data	Not reported	Not reported	Not reported	Not applicable	Three themes (1) Shift in self-perception (2) enhanced relationships (3) change in life outlook
Habib, Z.; Saddul, R.; Kamran, F., 2021 [38] Pakistan	To explore the perceptions and experiences of female burn survivors with Facial disfigurement in Pakistan.	*n* = 5 (all female)	Median 25 years (range 19–45 years)	Thematic analysis	Range 4–22 years	Not reported	Not reported	Not applicable	Physical appearance: Perceived stigmatization, self-perception and perception of others. Posttraumatic growth: sense of achievement, satisfaction and improved QoL. Acceptance, gratitude, optimism. Relationships: importance of good family support. Coping strategies: venting, religion, enduring.
Han, J.; Zhou, X.; Liu, J.; Yue, P.; Gao, L. 2020 [39] China	To explore resilience development in patients who have suffered a burn injury.	*n* = 10 (6 male)	Range = 19–44	Grounded theory	Not reported	Range 16–50%	Not reported		Five stages (1) black hole (2) introspection (3) integration (4) practice (5) growth Internal factors: Hope, sincerity, will, belief, curiosity. External factors: caring, support, sharing relationships. Ci = omitted relationships, and intimate relationships
Hunter, T.A.; Medved, M.I.; Hiebert-Murphy, D. Brockmeier, J.; Sareen, J.; Thakrar, S.; Logsetty, S.. 2013 [40] Canada	To contribute to a more complex understanding of survivors’ experiences through exploring the narratives and counter-narratives told by women who have experienced burn injury.	*n* = 10 (all female)	Mean = 45 Range 18–82	Narrative analytic method	Not reported	Mean = 8.75% Range 1–30%	Within 5 months of burn	Not applicable	The primary narratives were (1) “I don’t find it a problem” (2) not being ashamed to show others their scars and (3) not wanting to worry others.
Kornhaber, R.; Wilson, A.; Abu-Qamarm M.Z.; McLean, L. 2014 [42] Australia	To explore the concept from the lived experience about how burn survivors acknowledge and accept their burn injury.	*n* = 21 (20 male)	Average age 44y (range 21–65 years) (not stated if mean or median)	Qualitative phenomenological inquiry using semi-structured interviews and Colaizzi’s thematic analysis method.		Average TBSA 55% (range 20–90%) (not stated if mean or median)	6 months to 8 years	Not applicable	Acknowledgement (1) reasoning (gratefulness, downward comparison) (2) humour (3) the challenge of acceptance (4) self-awareness (confronting altered appearance)
Lau, U. & van Niekerk, A. 2011 [43] South Africa	To explore how young burn survivors define themselves and how the burn influences their worldview.	*n* = 6 (4 male)	Mean 19 years (range 14–24 years)	Interpretative social constructionism narrative approach	Not reported	Not reported	Not directly reported. >2 years postburn 3 as young children, 3 as (pre)adolescent	Not applicable	(1) The struggle for recognition—the self as both highly visible and invisible (2) Reconciling or rediscovering the self (3) Turning points: The search for meaning
Martin, L.; Byrnes, M.; McGarry, S.; Rea, S.; Wood, F. 2016 [44] Australia	To assess presentation of PTG after burn. To assess the use of the PTGI in burn patients.	*n* = 17 (64% male)	Median age Was 48 years (range 21–75 years)	Mixed method convergent parallel comparative approach. Qualitative semistructured interviews followed by comparison of previously collected quantitative PTGI responses.	Not reported	Median TBSA of 30% (range 15–85%)	Median Of 8 years post-burn (range 2–33 years)	Not applicable	Interpersonal relationships Trust and loyalty; Emotional transparency Independence vs dependence; Compassion Community support; Feelings of burden New possibilities Work-life balance; Recreation and leisure Citizenship (community contribution) Personal strength Gratefulness, planning, humour; Increased personal strength; Determination for independence Acceptance and will to move forward Spiritual change Used if existing faith only Appreciation of nature Better understanding of cycle of experiences (ie philosophical changes not spiritual changes) Appreciation of life Survival—gratitude; Wellbeing Accepting a new normal Use of time (life is short, live in the present) Value of relationships
Martin, L.; Byrnes, M.; McGarry, S.; Rea, S.; Wood, F. 2017 [45] Australia	To investigate adult burn survivors experience of visible scarring as barrier to PTG.	*n* = 16 (62% male)	Mean age of 46 years (*SD* 16.7; Range 18–61 years)	Qualitative phenomenological inquiry using semi-structured interviews using Tesch’s coding method.	Not reported	(TBSA) of 39.6 (*SD* 20.3; range 15–85%).	More than 2 years	Not applicable	Emotional barriers to growth (1) fear of rejection, (2) self-consciousness, and (3) embarrassment or humiliation. Situational barriers to growth (1) Inquisitive questions (2) obligation to explain Behavioural barriers to growth (1) Reactions of others (2) Pressure garments Coping strategies used Avoidant coping—avoidance of eye contact, closed body language etc. Active coping—humor, gratefulness, importance of relationships Discussion—risk of social isolation
McGarry, S.; Elliott, C.; McDonald, A.; Valentine, J.; Wood, F.; Girdler, S. 2014 [46] Australia	To explore the experience of children with burns.	*n* = 12 (6 male)	Range 8–15 years	Qualitative phenomenological inquiry using semi-structured interviews and Colaizzi’s thematic analysis method.		Range 1–20%	6 months	Not applicable	(1) the burn trauma (2) the recovery trauma Six themes (1) ongoing recurrent trauma; (2) returning to normal activities (3) behavioural changes; (4) scarring—permanent reminder; (5) family (6) adaptation—stronger, confidence, not letting small things bother them, resilience (or acceptance
McLean, L.M.; Rogers, V.; Kornhaber, R.; Proctor, M.; Kwietm J.; Streimer, J.; Vandervord, J. 2015 [47] Australia	To examine the early recovery lived experience for patients with a facial burn.	*n* = 6 (4 male)	Mean age 43 years (range 29–55 years)	Qualitative phenomenological inquiry using semi-structured interviews (Burns modified adult attachment interview) and Colaizzi’s thematic analysis method.		16.3% (0.8–55)	Interviewed within 4 months of burn [5 interviewed as inpatients, 1 within 4 months of injury LOS 20.3 days (range 3–60 days)]	Not applicable	Relationship to self/other—early self-image change and increased bodily awareness, change to interpersonal relationships (66%), altruism (100%). Coping—hopefulness about recovery (100%), positive rationalisation (66%), resilience, reflective appraisal, humour (100%). Meaning-making—retelling the tale, fear, panic, shock (83%), making sense of the accident (100%), history of previous trauma (100%), spirituality to find meaning (66%).
Moi, A.L. & Gjengedal, E. 2008 [48] Norway	To describe meanings in experience of life after Major burn injury.	*n* = 14 (11 male)	Mean 46 years (range 19–74)	Husserlian phenomenological Perspective, descriptive, Search for Meaning For a given context	Not reported	Mean TBSA 33% (range 7.5–62%)	Mean 14 m (range 5–35 m)	Not applicable	Facing the extreme—vigilance, action, need for assistance. A disrupted life history—creating coherence. Accepting the unchangeable—enduring, grief, fatalism, comparisons with others, and new feelings of gratefulness. Changing what is changeable—personal goals, independence, relationships with others, and a meaningful life, Regain freedom’
Neill, J.T.; Goch, I.; Sullivan, A.; Simons, M. 2021 [49] Australia	To explore the experience and longer term psychosocial impacts of burn camps.	*n* = 23 [patients *n* = 8. Parents *n* = 15 (subset of matched pairs *n* = 6)]	Median 11.2 year (range 8.1–14.9)	Inductive reflexive thematic analysis with pooled interview data from semistructured interviews with parents and children/adolescents	Birth—13 years (median 4.75 years)		Not stated	3 day burn camps	Camp experience (1) fun, adventurous activities, (2) social relatedness (3) camp setting and experience (4) acceptance Program outcomes (1) normalising experiences (2) social support (3) psychological recovery (4) confidence.
Williams, N.R.; Davey, M.; Klock-Powell, K. 2003 [50] USA	To explore the experience of recovery, and the influencing personal and environmental factors.	*n* = 7 (3 male)	Median age 40 years (range 31–52 years)	Phemenological analysis of semi-structured interviews	median age 32 years range 2–42 years		TBSA reported for 3 injuries, range 35–95%		Influences Construction of reality Time since injury Age when injured Themes Losses Gains/refaming Adaptation and coping with change Relationships with others
Zhai, J.; Liu, X.; Wu, J.; Jiang, H. 2010 [51] China	Do Chinese burn patients experience PTG? Are there PTG aspects not captured by PTGI? What common and unique factors facilitate PTG?	*n* = 10 (7 male)	Mean age 35y (range 24–48 years)	Qualitative, hermeneutic phenomenology, using semi-structured interviews		TBSA mean 69% (range 11–90)	Time since burn mean 2.8 years (range 5 months to 6.5 years)	Not applicable	PTG is ongoing process not a goal Process—need to manage emotions for cognitive processing to occur. Social system important. Effective coping style adequate abreaction, downward social comparison and seeking social support. For significant others— Meaning making Presentation of PTG Personal strength, new life philosophy; sharing of self; altruism born of suffering No spiritual religious growth reported by 90% of participant

## Data Availability

Data available on request to corresponding author.
